# Impact of additional selexipag on prostacyclin infusion analogs in patients with pulmonary arterial hypertension

**DOI:** 10.1016/j.rmcr.2022.101592

**Published:** 2022-01-30

**Authors:** Mizuki Momoi, Takahiro Hiraide, Yoshiki Shinya, Hiromi Momota, Shogo Fukui, Michiyuki Kawakami, Keiichi Fukuda, Masaharu Kataoka

**Affiliations:** a Department of Cardiology, Keio University School of Medicine, Tokyo, Japan; b Department of Rehabilitation, Keio University Hospital, Tokyo, Japan; c Department of Rehabilitation Medicine, Keio University School of Medicine, Tokyo, Japan; d Second Department of Internal Medicine, University of Occupational and Environmental Health, Kitakyushu, Japan

**Keywords:** Drug combinations, Selexipag, Prostacyclin

## Abstract

The effective therapy for pulmonary arterial hypertension (PAH) with inadequate clinical response is scarce except for lung transplantation when prostacyclin infusion is ineffective. The purpose of this study is to investigate the efficacy and safety of selexipag in addition to the infusion of prostacyclin. Nine patients [median 38 (36–49) years of age; 78% female] with PAH whose clinical response was inadequate despite the use of prostacyclin infusion analogs, were evaluated. Addition of selexipag significantly improved hemodynamics and no serious adverse events were observed. Selexipag with prostacyclin infusion analogs can be an effective therapeutic strategy for the PAH patients with inadequate clinical response.

## 1 Introduction

Pulmonary arterial hypertension (PAH) is a lethal cardiovascular disease. Currently, several types of oral pulmonary vasodilators and prostacyclin infusion analogs are available for PAH patients, and each drug has proven to be effective [[1], [2], [3], [4]]. However, when clinical responses are inadequate despite the use of prostacyclin infusion analogs, there is no alternative therapeutic strategy. Thus, the purpose of this study was to investigate the efficacy and safety of selexipag in addition to the infusion of prostacyclin or its analogs in PAH patients with inadequate clinical response.

## 2 Method

We retrospectively analyzed patients who were diagnosed with PAH at Keio University Hospital between 2016 and 2020 and received selexipag while being treated with prostacyclin infusion analogs (intravenous epoprostenol, intravenous treprostinil, or subcutaneous treprostinil). PAH was diagnosed in accordance with the current guidelines [5]. Selexipag was added when clinical responses were inadequate despite the use of prostacyclin infusion analogs and maximum tolerated dose of PAH-specific vasodilators targeting the endothelin pathway and nitric oxide pathway. The clinical response was assessed comprehensively using multiple indicators such as hemodynamics, World Health Organization functional class, 6-min walk distance (6MWD), and B-type natriuretic peptide (BNP) values according to the current guideline [5]. The hemodynamic state obtained by right heart catheterization, 6MWD, and BNP values were assessed at ∼6 months before selexipag use (time 1), just before the initiation of selexipag (time 2), and after the gradual increasing of selexipag doses (time 3). The events of all-cause death since time 2 were also collected. Regarding safety parameters, the maximum tolerated dose of selexipag and adverse events were investigated. Adverse events were defined as any undesirable medical complications associated with the addition of selexipag. These data were monitored until patients died or discontinued the use of selexipag or prostacyclin infusion analogs.

For statistical analyses, Friedman's test was used to compare the values at the 3 time points with *p* < 0.05 considered significant. If there were statistically significant differences, *post-hoc* analysis using Bonferroni's correction was performed, and *p*-value lowers than 0.017 were considered statistically significant. The analyses were conducted using SPSS software (version 25), and the Figure was generated using GraphPad Prism (version 8.0 for Mac; GraphPad Software, La Jolla, CA, USA). This study was approved by the institutional Ethics Committee, and written informed consent was obtained from all patients.

## 3 Result

Nine patients were included in this study. The baseline characteristics at time 1 were summarized in Table 1. Patients were predominantly female (78%) with a median age of 38 (interquartile range, 36–49) years at time 1. Three patients (33%) were diagnosed with idiopathic PAH, and 6 patients (67%) were heritable PAH. The median period from time 1 to time 2 and time 2 to time 3 was 150 days and 196 days, respectively.

**Table 1:** Patient demographics and characteristics at time 1.

Variable	Patients (n = 9)
Female, n (%)	7 (78%)
Age, years	38 (36–49)
WHO-FC, n (%)	
I/II/III/IV	0/7/2/0 (0%/78%/22%/0%)
BNP, pg/mL	19 (8–25)
6MWD, m	420 (293–540)
Hemodynamics	
mean RAP, mmHg	5 (4–7)
mean PAP, mmHg	42 (36–56)
PAWP, mmHg	7 (7–8)
CO, L/min	5.0 (4.1–5.9)
PVR, WU	7.4 (5.4–10.9)
PAH etiology, n (%)	
IPAH	3 (33%)
HPAH	6 (67%)
Genetic mutations or deletions^a^, n (%)	
*BMPR2*	5 (56%)
*SOX17*	1 (11%)

Data are expressed as number (%) or median (interquartile range).

a All patients were genetically tested, and 3 had no genetic mutations or deletions related to PAH. *BMPR2*, bone morphogenetic protein receptor type 2; BNP, B-type natriuretic peptide; CO, cardiac output; HPAH, heritable pulmonary arterial hypertension; IPAH, idiopathic pulmonary arterial hypertension; PAH, pulmonary arterial hypertension; PAP, pulmonary arterial pressure; PAWP, pulmonary arterial wedge pressure; PVR, pulmonary vascular resistance; RAP, right atrial pressure; *SOX17*, SRY-box 17; WHO-FC, World Health Organization functional class; WU, Wood units; 6MWD, 6-min walk distance.

**Fig. 1:**
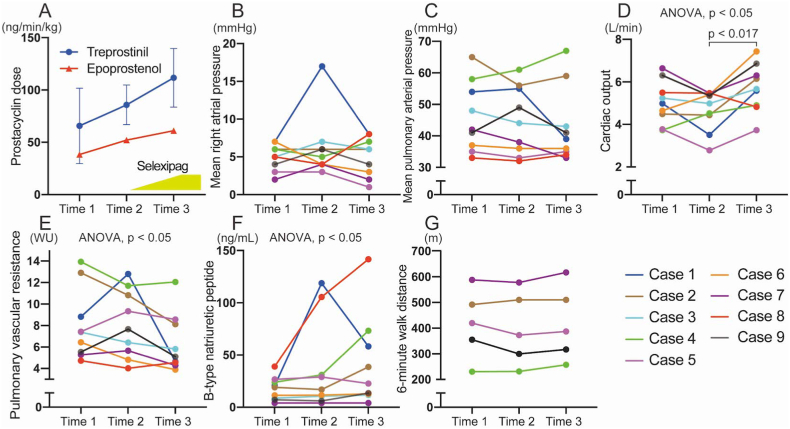
**Changes in prostacyclin doses and clinical parameters of patients with pulmonary arterial hypertension.** The changes in the dose of prostacyclin (**A**), hemodynamics obtained from right heart catheterization (**B–E**), B-type natriuretic peptide (**F**), and 6-min walk distance (**G**) at the 3 time points, including ∼6 months before selexipag use (time 1), just before the initiation of selexipag (time 2), and after the gradual increasing of selexipag doses (time 3). Clinical parameters were measured in 9 patients, but the 6-min walk distance was assessed in 5 patients. ANOVA, Analysis of variance; WU, Wood units.

The mean prostacyclin dose at each time point was shown in Fig. 1-A. At time 1, 3 patients received an intravenous infusion of epoprostenol, 1 patient received an intravenous infusion of treprostinil, and 4 patients received a subcutaneous infusion of treprostinil. At time 2 and time 3, another patient received an intravenous infusion of treprostinil. The changes in prostacyclin doses are shown in Fig. 1-A. The mean selexipag dose at time 3 was 2.8 ± 0.47 mg/day. Although the mean PAP did not significantly change [42 (36–56) mmHg at time 1, 44 (35–56) mmHg at time 2, and 39 (35–51) mmHg at time 3] (Fig. 1-C), the cardiac output significantly improved after the addition of selexipag [5.0 (4.1–5.9) L/min at time 1, 5.0 (4.0–5.4) L/min at time 2, and 5.7 (4.9–6.6) L/min at time 3] (time 2 vs. time 3, *p* = 0.015 with *post-hoc* analysis) (Fig. 1-D). PVR [7.4 (5.4–10.9) Wood units at time 1, 7.7 (5.2–11.3) Wood units at time 2, and 5.1 (4.4–8.4) Wood units at time 3] and BNP [19 (8–25) pg/mL at time 1, 17 (8–68) pg/mL at time 2, and 23 (12–66) pg/mL at time 3] significantly changed (*p* = 0.045 and *p* = 0.044 with Friedman's test, respectively) (Fig. 1-E and Fig. 1-F). The mean right atrial pressure and 6MWD were not significantly different among the 3 time points (Fig. 1-B and Fig. 1-G). During a follow-up period [46 (43–54) months], a total of two patients died: one due to heart failure and one due to pneumonia.

Regarding the safety analysis, patients experienced diarrhea (56%), nausea (44%), headache (33%), and jaw pain (22%). Muscle aches, fatigue, dizziness, rash, and pain in extremities occurred in 11% of patients. No serious adverse events, including death, hospitalization, or life-threatening adverse drug reactions, were observed. Although the selexipag dose was maintained at 2.4 mg/day due to nausea in only one patient, the other patients (89%) continued selexipag at the maximum tolerated dose of 3.2 mg/day.

Among 9 patients, addition of selexipag was highly effective in the case 1 (Fig. 1). This patient was diagnosed with idiopathic PAH at 35 years old and the mean PAP, PVR, and cardiac output was 54 mmHg, 8.82 Wood units, and 4.99 L/min, respectively. Subcutaneous injection of treprostinil in addition to the oral pulmonary vasodilators with macitentan and tadalafil was initiated and up-titrated to 67 ng/kg/min. However, her hemodynamics did not improve at all (mean PAP: 55 mmHg, PVR: 12.8 Wood units, cardiac output: 3.51 L/min at time 2). Selexipag was added to the subcutaneous treprostinil, and her hemodynamics improved drastically after 6 months (mean PAP: 39 mmHg, PVR: 4.65 Wood units, cardiac output: 5.59 L/min at time 3). The dose of treprostinil and selexipag was 70 ng/kg/min and 2.8 mg/day, respectively. Furthermore, in the case 9, although the dose of intravenous epoprostenol was slightly up-titrated (40 ng/kg/min at time 1, 60 ng/kg/min at time 2, and 76 ng/kg/min at time 3), mean PAP and PVR was improved after addition of selexipag (mean PAP: 41 mmHg at time 1, 49 mmHg at time 2, and 41 mmHg at time 3; PVR: 5.5 Wood units at time 1, 7.7 Wood units at time 2, and 5.1 Wood units at time 3). Conversely, the effect of additional selexipag was not observed in the case 4. In this case, clinical response of the patient with idiopathic PAH was inadequate despite the use of intravenous treprostinil (100 ng/kg/min at time 2), macitentan, and tadalafil. The mean PAP, PVR, and cardiac output was 61 mmHg, 11.7 Wood units, and 4.53 L/min, respectively. Although the selexipag was initiated together with further up-titration of intravenous treprostinil, her hemodynamics did not improve after 6 months (mean PAP: 67 mmHg, PVR: 12.05 Wood units, cardiac output: 4.90 L/min at time 3). After 3 years, she died of heart failure despite further up-titration of intravenous treprostinil. Any adverse events associated with initiation of selexipag were not observed in these cases. Verification to predict how patients are effective with additional selexipag on prostacyclin infusion analogs, will be needed in the future.

## 4 Discussion

In this study, we investigated the effectiveness of selexipag combined with prostacyclin infusion analogs in PAH patients. Although there have been several reports in which prostacyclin infusion analogs were switched to selexipag [6,7], no clinical studies have investigated the efficacy of selexipag combined with prostacyclin infusion analogs. The hemodynamic results in this study suggested that selexipag showed additional favorable effects in PAH patients with inadequate clinical response even after receiving prostacyclin infusion analogs, although the effect of prostacyclin titration could not be excluded. The metabolic product of selexipag ACT-333679 functions as a prostacyclin receptor agonist [8]. Selexipag is less likely to cause desensitization due to internalization of the prostacyclin receptor than prostacyclin infusion analogs [8]. Therefore, considering the results in this study, the addition of selexipag provides a potential strategy for patients with inadequate clinical response. Regarding the safety analysis, the frequency of adverse events after the addition of selexipag in this study was equivalent to that in a previous study without prostacyclin infusion [1], suggesting the permitted tolerability of selexipag combined with prostacyclin infusion.

This study had several limitations, including the small number of patients and retrospective single-center design. Furthermore, the dose and types of prostacyclin analogs and other oral medications varied in each patient and at each time point. Particularly, the influence by dose of the prostacyclin infusion analogs should be carefully taken into consideration, as it was up-titrated sequentially in many cases.

In conclusion, although further studies are needed to validate the efficacy, selexipag in addition to prostacyclin infusion analogs can be an effective therapeutic strategy for PAH patients whose clinical responses are inadequate despite the use of prostacyclin infusion analogs.

## Data availability

The deidentified participant data will not be shared.

## IRB information

This study was approved by the Keio university institutional Ethics Committee (approval No. 20140203) and written informed consent was obtained from all patients.

## Declaration of competing interest

There are no sources of support in the form of grants, gifts, equipment, and/or drugs. There are no conflicts of interest associated with this study.
